# Structural Features of Sulfated Glucuronomannan Oligosaccharides and Their Antioxidant Activity

**DOI:** 10.3390/md16090291

**Published:** 2018-08-21

**Authors:** Weihua Jin, Langlang Ren, Bing Liu, Quanbin Zhang, Weihong Zhong

**Affiliations:** 1 College of Biotechnology and Bioengineering, Zhejiang University of Technology, Hangzhou 310023, China; jinweihual@zjut.edu.cn (W.J.); 15700084451@163.com (L.R.); liubing19950108@163.com (B.L.); 2 Laboratory of Experimental Marine Biology, Institute of Oceanology, Chinese Academy of Sciences, Qingdao 266071, China; 3 Center for Ocean Mega-Science, Chinese Academy of Sciences, Qingdao 266000, China

**Keywords:** glucuronomannan oligosaccharides, sulfation, antioxidant activity

## Abstract

Glucuronomannan oligosaccharides (Gs) were derived from fucoidan, which was extracted from the brown alga *Sargassum thunbergii*. Sulfated glucuronomannan oligosaccharides (SGs) were obtained by the sulfation of Gs. NMR techniques were used to reveal that the order of sulfation was Man-C6 > Man-C4 > Man-C1R > GlcA-C3 > Man-C3 > GlcA-C2. Finally, the antioxidant activities (hydroxyl radical scavenging activity, superoxide radical scavenging activity, reducing power and DPPH radical scavenging activity) of Gs and SGs were determined. The findings showed that the higher the degree of polymerization, the better the activity, except for the hydroxyl radical scavenging activity. In addition, the higher the sulfate content, the lower the activities for the reducing power and DPPH radical scavenging activity. Opposite results were found for the superoxide radical scavenging activity. Finally, compared with fucoidan, most Gs and SGs had higher antioxidant activity, suggesting that they might be good candidates for antioxidants.

## 1. Introduction

Oligosaccharides play crucial roles in a wide range of biological processes, such as cellular communication, pathogenesis and prebiotic functions [1,2,3,4,5]. For this reason, they are also being developed for new drugs. In addition, they have advantages over polysaccharides, which cannot pass through barriers in the body, even cell membrane. Thus, more research on oligosaccharides is being conducted.

In this study, glucuronomannan oligosaccharides obtained from degradation of fucoidan that was extracted from the brown alga *Sargassum thunbergii*. Glucuronomannan is one of the main backbones of fucoidan, which had a variety of biological activities, including antioxidant, antitumour and neuroprotective activities [6,7,8,9,10,11]. In addition, glucuronomannan has many branches, including sulfate group, fucopyranoside and fucooligopyranoside [12,13,14]. However, there has been little research on the activities of glucuronomannan oligosaccharides or sulfated glucuronomannan oligosaccharides.

Sulfation is important since it changes a polysaccharides’ antioxidant, antiviral, antitumour activities, etc. [15,16,17,18]. It was found that the inhibitory effects of fucans on both coagulation and cell proliferation decreased with decreasing sulfate content [15]. It was found that oversulfated fucoidan reduced the proliferation of U937 cells in a dose-dependent manner whereas native fucoidan showed no such activity [19]. Chevolot et al. [20] reported that anticoagulant activities of fucoidan were also dependent on sulfate contents.

Thus, we tried to elucidate the patterns of sulfation using the sulfur trioxide-pyridine method in this study. In addition, we also tried to illustrate the effects of sulfate content and degree of polymerization on antioxidant activities.

## 2. Results 

### 2.1. Preparation of Sulfated Glucuronomannan Oligosaccharides

By changing the molar ratios of glucuronomannan oligosaccharides (G2, G4 and G6) to sulfur trioxide-pyridine, glucuronomannan oligosaccharides with different degree of sulfation were obtained. Then, the amounts were confirmed by ESI-MS. The results (Figures S1–S8) are summarized in Table 1. It was shown that G2 had two different fractions (G2S1, which was GlcAMan(SO_3_H)_3–6_ with high degree of sulfation, and G2S2, which was GlcAMan(SO_3_H)_1–3_ with low degree of sulfation). G4 had three different fractions (G4S1, which was GlcA_2_Man_2_(SO_3_H)_8–11_ with high degree of sulfation, G4S2, which was GlcA_2_Man_2_(SO3H)_5–9_ with medium degree of sulfation, and G4S3, which was GlcA_2_Man_2_(SO3H)_1–5_ with low degree of sulfation). G6 had three different fractions (G6S1, which was GlcA_3_Man_3_(SO3H)_8–15_ with high degree of sulfation, G6S2, which was GlcA_3_Man_3_(SO3H)_4–10_ with medium degree of sulfation, and G6S3, which was GlcA_3_Man_3_(SO3H)_1–6_ with low degree of sulfation).

### 2.2. Structural Analysis of Sulfated Glucuronomannan Oligosaccharides by ESI-CID-MS/MS

**Figure 1:**
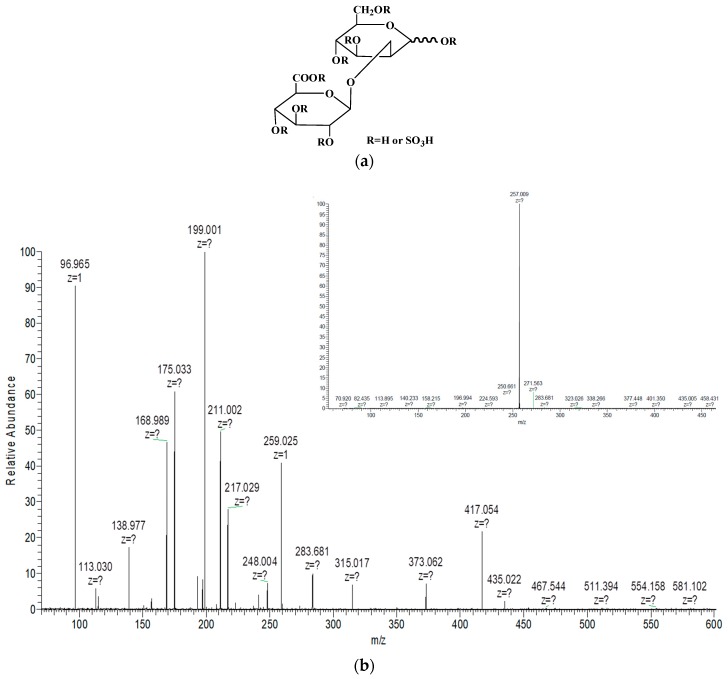
(**a**) The chemical structure of disaccharide GlcAMan(SO_3_H)_3_. (**b**) Negative-ion mode electrospray ionization in tandem with collision-induced dissociation tandem mass (ESI-CID-MS/MS) spectrum of the ion at *m*/*z* 296.991 (-2), corresponding to GlcAMan(SO_3_H)_3_ and ESI-CID-MS/MS/MS spectrum of the ion at *m*/*z* 257.009 (-2), corresponding to GlcAMan(SO_3_H)_2_.

To elucidate the structural features of sulfated glucuronomannan oligosaccharides, ESI-CID-MS/MS was performed. The ESI-CID-MS/MS spectrum of the ion at *m*/*z* 296.991 (-2) ([GlcAMan(SO_3_H)_3_-2H]^2−^) is shown in Figure 1. Only one ion at *m*/*z* 257.009 (-2), corresponding to [GlcAMan(SO_3_H)_2_-2H]^2−^, was derived from the loss of sulfur trioxide (−80 Da). MS/MS/MS (Figure 1) was performed to confirm the sulfation pattern. The ions at *m*/*z* 435.022 (-1) and 217.029 (-2) were also derived from the loss of sulfur trioxide (−80 Da), which was confirmed by the presence of an ion at *m*/*z* 96.965. The absence of an ion at *m*/*z* 355 [GlcAMan-H]^−^ proved that the last sulfate group was relatively stable. The ion at *m*/*z* 259.025 indicated that the sulfate group was substituted at the Man residue, which was confirmed by the presence of an ion at *m*/*z* 175.033 (Y_1_-type ion), suggesting that the GlcA residue was not sulfated. The characteristic ion at *m*/*z* 199.001 (^0,2^X-type ion) indicated that the linkage of the GlcA residue and the Man residue was 1→2, which was consistent with a previous study [13]. On the other hand, it also indicated that the sulfation group was substituted at C4 or C6 of the Man residue, because the formation of ^0,2^X originated from the C3 hydroxy group [21]. In addition, the presence of fragment ions at *m*/*z* 168.989 (^0,3^X-type ion) and 138.977 (^0,4^X-type ion) indicated that the sulfation was at C6 of the Man residue. No ions at *m*/*z* 255 and 273 were found. Thus, it was concluded that it was difficult to determine the precise location of sulfation.

**Figure 2:**
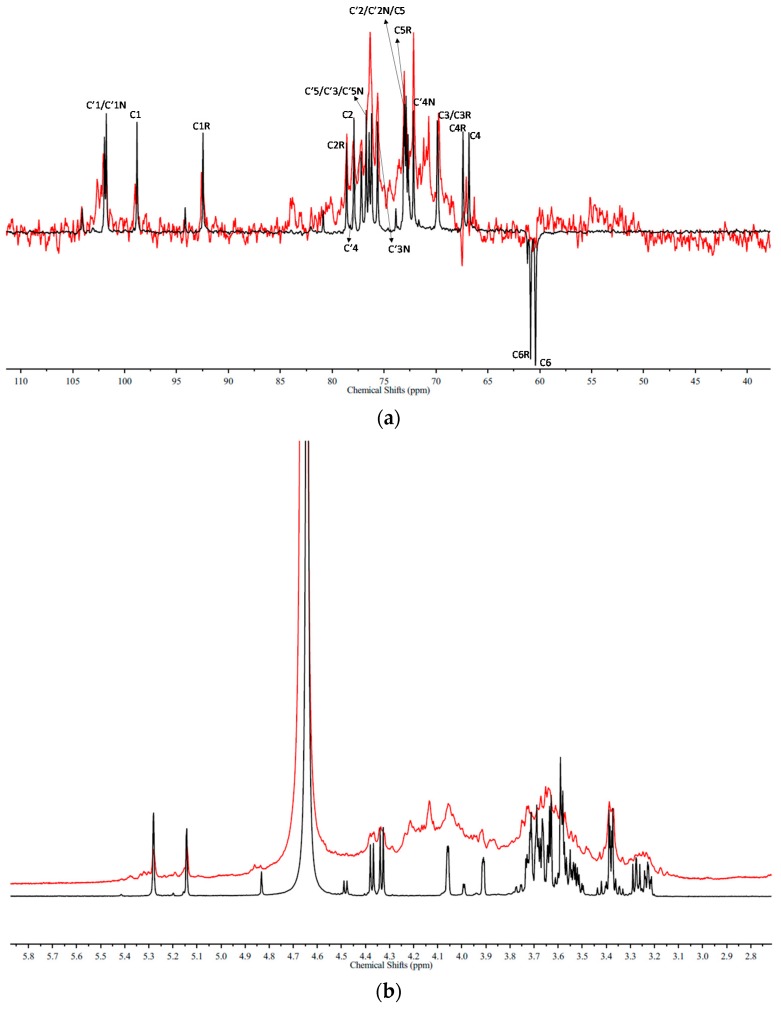
(**a**) The DEPTQ spectra and (**b**) ^1^H-NMR spectra of a glucuronomannan-tetramer (G4) and its low sulfated fraction (G4S3) (Red).

**Figure 3:**
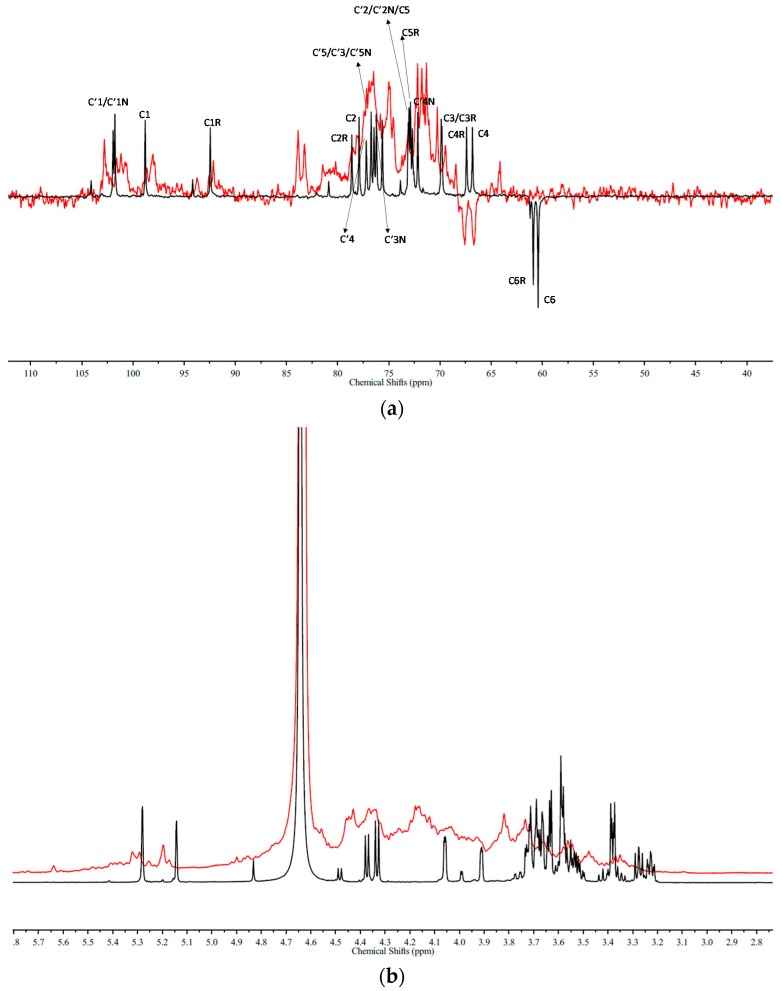
(**a**) The DEPTQ spectra and (**b**) 1H-NMR spectra of a glucuronomannan-tetramer (G4) and its medium sulfated fraction (G4S2) (Red).

Finally, NMR was performed to elucidate the precise location of sulfation. Figure 2 shows the DEPTQ spectra of the glucuronomannantetramer (G4) and its low sulfated fraction (G4S3). The chemical shifts of G4 and G6 were shown in Table S1. Comparison of these two DEPTQ spectra shows that: (1) The reversal peaks of the Man residue C6 at the reducing end (C6R) and the non-reducing end (C6) were missing, while another new reversal peak with a chemical shift of 67.5 ppm was found (another reversal peak was overlapped, which was confirmed by Figure 3), suggesting that the Man residue at C6 was sulphated. (2) The intensity of the peaks of the Man residues C4 at the non-reducing end (C4) and the reducing end (C4R) decreased, indicating that the Man residues C4 at the non-reducing end (C4) and the reducing end (C4R) were partially sulphated. (3) The intensity of the peak of the Man residue C1 at the reducing end (C1R) was relatively unmodified, indicating that the Man residue C1 at the reducing (C1R) was not sulfated. To further confirm the precise location of sulfation, we compared the ^1^H-NMR spectra of the glucuronomannantetramer (G4), and its low sulfated fraction (G4S3) (Figure 2). It was shown that (1) the chemical shifts of the Man residue H1 at the reducing end (5.14 ppm) (H1R) and the non-reducing end (5.28 ppm) (H1) and the GlcA residue H1 at the reducing (4.37 ppm) (H’1) and the non-reducing end (4.33 ppm) (H’1N) were the same, indicating that the Man residues C3 at the reducing end (C3R) and the non-reducing end (C3) and the GlcA residues C2 at the reducing end (C’2) and the non-reducing end (C’2N) were not sulfated. These findings were also confirmed by the presence of the Man residues H2 at the reducing (3.91 ppm) (H2R) and the non-reducing ends (4.05 ppm) (H2) and the GlcA residue H2 at the reducing (3.23 ppm) (H’2) and the non-reducing ends (3.28 ppm) (H’2N); (2) In addition, the chemical shift of the GlcA residue H3 at the non-reducing end (3.38 ppm) (H’3N) did not change, indicating that it was not sulfated. Moreover, the signal of GlcA_2_Man_2_(SO_3_H)_5_ was weak in the MS spectrum. In conclusion, the findings suggest that the order of sulfation might be Man-C6 ≈ Man-C6R > Man-C4 ≈ Man-C4R.

The DEPTQ and ^1^H-NMR spectra of glucuronomannan-tetramer (G4) and its medium sulfation fraction (G4S2) were shown in Figure 3. A comparison of these two DEPTQ spectra (Figure 3a) shows that: (1) The reversal peaks of the Man residues C6 at the reducing (C6R) and the non-reducing end (C6) were missing, while another two new reversal peaks with chemical shifts of 67.6 ppm and 66.7 ppm appeared in the spectrum, suggesting that the Man residues at C6 were both sulfated. (2) The peaks of the Man residues C4 at the non-reducing end (C4) and the reducing end (C4R) were missing, indicating that the Man residues of C4 at the non-reducing end (C4) and the reducing end (C4R) were sulfated. (3) The peak of the Man residue C1 at the reducing (C1R) was missing, indicating that the Man residue of C1 at the reducing (C1R) was sulfated. (4) There were three peaks with chemical shifts of 100.6 ppm, 101.2 ppm and 101.6 ppm, which were influenced by the sulfation of the GlcA residues C3 at the reducing end (C’3) and the non-reducing end (C’3N). (5) Comparison with the DEPTQ spectra of G4S3 and G4S2 (Figure S9 in the Supplementary Materials), it was found that the Man residues C3 at the reducing end (C3R) and the non-reducing end (C3) were missing, indicating that they were sulfated. To further confirm the precise location of sulfation, the ^1^H-NMR spectra of the glucuronomannan-tetramer (G4) and its medium sulfation fraction (G4S3) were compared (Figure 3b). These ^1^H-NMR spectra show that: (1) The peak of the Man residue H1 at the reducing end (5.14 ppm) (H1R) was missing, indicating that it was sulfated, which was also confirmed by a new peak with a chemical shift of 5.64 ppm. (2) The chemical shifts of the GlcA residues H1 at the non-reducing end (H’1N) and the reducing end (H’1) were unchanged. In addition, the peaks of the GlcA residues H2 at the non-reducing end (H’2N) and the reducing end (H’2) were missing. Thus, it was suggested that the GlcA residues H3 at the non-reducing end (H’3N) and the reducing end (H’3) were sulfated. (3) There were no peaks of the Man residues H2 at the non-reducing end (4.05 ppm) (H2) and the reducing end (3.91 ppm) (H2R), which were influenced by the sulfation of the Man residues H1 at the reducing end (H1R) and H3 at the reducing end (C3R) and the non-reducing end (C3). Consequently, it was concluded that the order of sulfation might be Man-C6 ≈ Man-C6R > Man-C4 ≈ Man-C4R > Man-C1R > GlcA-C’3N ≈ GlcA-C’3 > Man-C3 ≈ Man-C3R.

**Figure 4:**
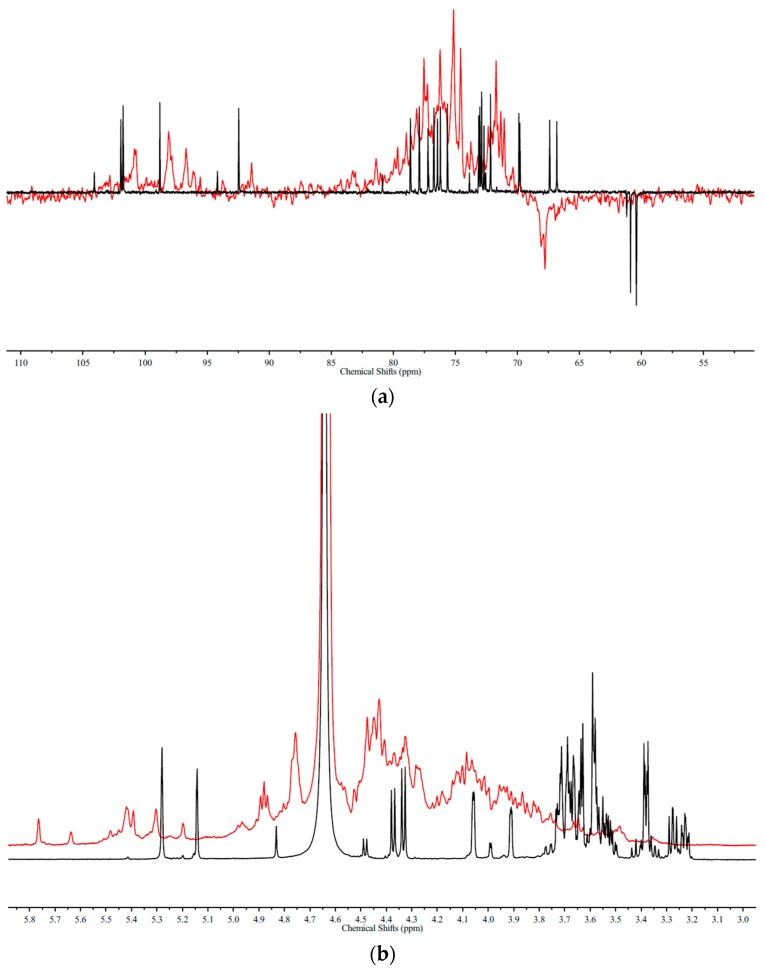
(**a**) The DEPTQ spectra and (**b**) 1H-NMR spectra of a glucuronomannan-tetramer (G4) and its high sulfated fraction (G4S1) (Red).

Figure 4 are the DEPTQ (a) and ^1^H-NMR (b) spectra of glucuronomannan-tetramer (G4) and its high sulfation fraction (G4S1). The spectra were relatively complicated. However, it was proposed that the order of sulfation might be Man-C6 ≈ Man-C6R > GlcA-C’4N > Man-C4 ≈ Man-C4R > Man-C1R > GlcA-C’3N ≈ GlcA-C’3 > Man-C3 ≈ Man-C3R > GlcA-C’2N ≈ GlcA-C’2, based on the above results.

Finally, we propose that the order of sulfation on glucuronomannan-oligomers is Man-C6 > GlcA-C4 > Man-C4 > Man-C1R > GlcA-C3 > Man-C3 > GlcA-C2.

### 2.3. Antioxidant Activities of Glucuronomannan Oligosaccharides and Their Sulfated Fractions

Four different assays (hydroxyl radical scavenging activity, superoxide radical scavenging activity, reducing power and DPPH radical scavenging activity) were conducted to investigate the effects of sulfate content and the degree of polymerization on antioxidant activities.

**Figure 5:**
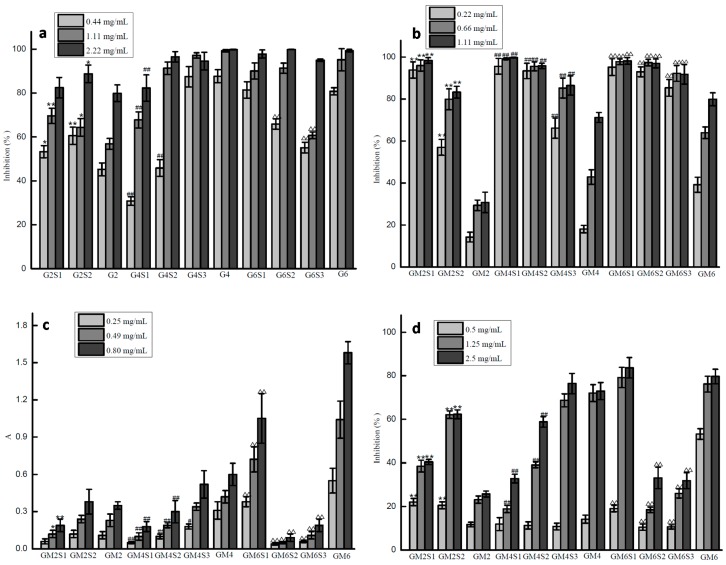
The scavenging effects of different glucuronomannan oligosaccharides and their sulfated fractions on hydroxyl radical (**a**), superoxide radical (**b**), reducing power (**c**) and DPPH free radical (**d**). Values are expressed as the mean ± SD from three replicates. (* *p* < 0.05 compared to the G2; ** *p* < 0.01 compared to the G2; # *p* < 0.05 compared to the G4; ## *p* < 0.01 compared to the G4; Δ *p* < 0.05 compared to the G6; ΔΔ *p* < 0.01 compared to the G6).

The hydroxyl radical scavenging assay (Figure 5A) demonstrated the ability of the samples to scavenge hydroxyl radical produced by the Fenton system. This assay showed that most samples exhibited hydroxyl radical scavenging activity in a concentration-dependent manner. Comparison with glucuronomannan oligosaccharides (G2, G4 and G6), G4 was the best, followed by G6, and G2 has the least hydroxyl radical scavenging activity. In terms of sulfate content, the activity was dependent on the degree of polymerization. The activity for G6 and its fractions was in the following order: G6 > G6S1 > G6S2 > G6S3, the activity for G4 and its fractions was G4 > G4S3 > G4S2 > G4S1, and the activity for G2 and its fractions was G2S2 > G2S1 > G2. Thus, we concluded that G4 and G4S3 showed the best activity, while fucoidan (the raw material of glucuronomannan oligosaccharides) showed only 30% activity at a concentration of 7 mg/mL [22]. Previous studies [23,24] have shown that there are two mechanisms responsible for scavenging hydroxyl radical: First is to inhibit the production of hydroxyl radical, and another is to remove the hydroxyl radical generated. The first mechanism is correlated with the ability of chelating metal ions, which depends on molecular weight, sulfate content and uronic acid content. Thus, we proposed that the activity of glucuronomannan oligosaccharides and their sulfated fractions was influenced by the sulfate content and the degree of polymerization in inhibiting the production of hydroxyl radical.

Although superoxide radical is a weak oxidant in most organisms, it can also be degraded continuously to produce new active reactive oxygen species (ROS), leading indirectly to the peroxidation of lipids and directly causing diseases such as arthritis and Alzheimer’s disease [25,26]. For this reason, it was essential to investigate the effects of the sulfate content and the degree of polymerization on superoxide radical scavenging activity. Information on these effects might help to screen for novel drugs. Figure 5B shows that a positive correlation exists between superoxide radical scavenging activity and the concentration of most samples. When compared with sulfated glucuronomannan oligosaccharides, the glucuronomannan oligosaccharides showed lower scavenging activity, indicating that sulfate was important for this activity. In addition, the higher the sulfate content, the better the superoxide radical scavenging activity. Similarly, the higher the degree of polymerization, the better the activity. When compared with fucoidan, whose activity was approximately 80% at a concentration of 50 μg/mL [22], glucuronomannan oligosaccharides and their sulfated fractions had lower superoxide radical scavenging activity.

The reducing power assay determines the reducing power using Fe^3+^/ferricyanide and furnished an important index of potential antioxidant activity. Figure 5C shows that the reducing power increased with increasing concentration, displaying a linear correlation. Compared with glucuronomannan oligosaccharides, the result showed that the higher the degree of polymerization, the better the reducing-power. In terms of sulfate content, the results show that sulfated glucuronomannan oligosaccharides had lower reducing power. This phenomenon was opposite to the superoxide radical scavenging activity, except for G6S1. When compared with fucoidan (with an absorbance of approximately 0.1 at a concentration of 2.5 mg/mL) [22], glucuronomannan oligosaccharides and their sulfated fractions showed higher reducing power.

Compared with other methods of evaluating antioxidant activities, the DPPH radical scavenging assay is a time-saving and rapid method and it is widely used for this reason. Most samples exhibited a positive correlation between the measured activity and the concentration of the polysaccharides (Figure 5D). When compared with glucuronomannan oligosaccharides, it was shown that the higher the degree of polymerization, the better the activity. In terms of the sulfate content, it was found that sulfated glucuronomannan oligosaccharides had lower DPPH radical scavenging activity. This phenomenon was the same with regards to reducing power but was opposite to superoxide radical scavenging activity, except for G6S1. When compared with low molecular weight fucoidan [27], whose activity was approximately 80% at a concentration of 7.0 mg/mL, glucuronomannan oligosaccharides and their sulfated fractions have higher activity.

## 3. Discussion

Glucuronomannan oligosaccharides (Gs) were derived from fucoidan, which was extracted from the brown alga *Sargassum thunbergii*. Sulfated glucuronomannan oligosaccharides (SGs) were obtained by the sulfation of Gs. Ten samples with different sulfate content and degree of polymerization were prepared to elucidate the structure-activity relationships between sulfated oligosaccharides and antioxidant activities, including hydroxyl radical scavenging activity, superoxide radical scavenging activity, reducing power and DPPH radical scavenging activity. It was shown that: (1) The glucuronomannan tetramer (G4) and the glucuronomannan hexamer (G6) had higher hydroxyl radical scavenging activities than their partial sulfate fractions and the glucuronomannan dimer (G2), while the activity of the glucuronomannan dimer (G2) was lower than that of its sulfated fractions at the concentrations of 0.44 mg/mL and 1.11 mg/mL. Therefore, there was no correlation between sulfate content and degree of polymerization and hydroxyl radical scavenging activity. (2) With regards to the superoxide radical scavenging activity, the results show that the higher the degree of polymerization, the better the activity. Similarly, the higher the sulfate content, the better the activity. (3) For the reducing power and DPPH radical scavenging activities, similar trends of the superoxide radical scavenging activity occurred. The results also show that the higher the degree of polymerization, the better the activity. On the contrary, the higher the sulfate content, the lower the activity, except for G6S1. Therefore, it was concluded that the structure-activity relationship for different activities were different. In another word, there was no absolute correlation suitable for all structure-activity relationship of Gs and SGs. Further studies on the neuroprotective activity of sulfated oligosaccharides will be determined to elucidate their structure-activity relationship.

## 4. Materials and Methods

### 4.1. Preparation of Glucuronomannan Oligosaccharides

The brown alga *Sargassum thunbergii* was collected in Qingdao, China. The alga (100 g) was cut into pieces. Crude polysaccharide from *Sargassum thunbergii* was extracted with hot water (3 L) for 4 h. The aqueous solution was filtered with Celite and concentrated by rotary evaporator. Elimination of alginate was achieved using 20% ethanol in water with MgCl_2_. After removing the alginate, the supernatant was dialyzed, concentrated and precipiteted by ethanol. Glucuronomannan oligosaccharides were prepared according to a protocol modified from a previous study [13]. Briefly, crude polysaccharide was refluxed in 4% sulfuric acid aqueous solution (60 mg/mL) for 5 h and then neutralized with barium hydroxide after cooling to room temperature. The solution was centrifuged, and the supernatant was concentrated by rotary evaporator. The concentrated solution was fractionated using an activated carbon column (2.6 cm × 30 cm) with water (Y1) and 50% ethanol (Y2). Then, eluent Y2 was combined, concentrated and freeze-dried. Y2 (0.5 g) was separated on a Bio-Gel P-4 Gel column (Extra Fine, <45 μM, 2.6 cm × 100 cm) and then eluted with 0.5 M NH_4_HCO_3_ at a flow rate of 0.15 mL/min. Fractions were collected every 14 min per pipe after 27 h. Glucuronomannan oligosaccharides (G2 stands for glucuronomannan dimer, G4 stands for glucuronomannan tetramer, and G6 stands for glucuronomannan hexamer) were collected and lyophilized. The yields of G2, G4 and G6 were 45.9%, 23.6% and 10.4%, respectively.

### 4.2. Preparation of Sulfated Glucuronomannan Oligosaccharides

The sulfated glucuronomannan oligosaccharides were prepared according to a protocol modified from the previous study [28]. Oligomers (G2, G4 and G6) with different sulfur trioxide-pyridine were added to dimethylformamide (DMF) at room temperature for 24 h, after which the solution was diluted, neutralized and concentrated. Then, the solution was desalted on a Sephadex G-10 column (5 cm × 50 cm), and eluted with water. Finally, the sulfated glucuronomannan oligosaccharides were purified on a Bio-Gel P-4 Gel column (Extra Fine, <45 μM, 2.6 cm × 100 cm) and eluted with 0.5 M NH_4_HCO_3_ at a flow rate of 0.15 mL/min.

### 4.3. MS Analysis of Oligosaccharides

ESI-MS and ESI-CID-MS/MS were performed on an LTQ ORBITRAP XL (Thermo Scientific, Waltham, MA, USA). The samples were dissolved in CH_3_CN-H_2_O (1:1, *v*/*v*). The solution was centrifuged, and the supernatant was analyzed. Mass spectra were registered in a negative ion mode at a flow rate of 5 μL/min. The capillary voltage was set to −3000 V, and the cone voltage was set at −50 V. The source temperature was 80 °C, and the desolvation temperature was 150 °C. The collision energy was optimized between 10 and 50 eV. All spectra were analyzed by Xcalibur.

### 4.4. NMR Spectroscopy

Oligosaccharides (20 mg) were co-evaporated with deuterium oxide (99.9%) twice before dissolving in deuterium oxide (99.9%). 1D and 2D NMR spectra were recorded on a Bruker AVANCE III (Bruker BioSpin, Billerica, MA, USA) at 600 MHz and 25 °C.

### 4.5. Investigation of Antioxidant Activity

#### 4.5.1. The Activity of Scavenging Hydroxyl Radicals

The ability of the samples to scavenge hydroxyl radical was determined according to a modified method [27]. Briefly, 2 mmol/L EDTA-Fe (0.5 mL), sodium phosphate buffer (150 mM, pH = 7.4, 1 mL), Safranine T (dissolved in above sodium phosphate buffer, 1 mL), and 3% H_2_O_2_ (1 mL) were added sequentially into the solution (1 mL) with various concentrations of samples (First, the original concentrations of all samples were 25 mg/mL. Then, they were diluted with different buffers). Then, the mixtures were incubated at 37 °C for 30 min, and the absorbance at 520 nm was measured. In the control, distilled water was substituted for sample and sodium phosphate buffer was substituted for H_2_O_2_. The scavenging effect (percentage) was calculated using the following equation: Scavenging effect (%) = A_sample_/A_control_ × 100.

#### 4.5.2. The Activity of Scavenging Superoxide Radicals

A superoxide radical assay was conducted according to a modified method [27]. Briefly, 0.5 mL nicotinamide adenine dinucleotide-reduced (NADH) (0.0365%, *w*/*v*), 0.5 mL nitro blue tetrazolium (NBT) (0.0246%, *w*/*v*), and 0.5 mL phenazine methosulfate (PMS) (0.002%, *w*/*v*) were added sequentially into Tris-HCl buffer (16 mmol/L, pH = 8.0) (3 mL) with different concentrations of samples (First, the original concentrations of all samples were 25 mg/mL. Then, they were diluted with different buffers). Then, the absorbance of the solutions at 560 nm was measured. In controls, Tris-HCl buffer was substituted for sample. The scavenging effect (percentage) was calculated using the following equation: Scavenging effect (%) = (1 − A_sample_/A_control_) × 100.

#### 4.5.3. The Reducing Power Assay

The reducing power assay was conducted according to a modified method [29]. Briefly, 1.25 mL of potassium ferricyanide (1%, *w*/*v*) was added to the solution (1 mL) with different concentrations of samples (First, the original concentrations of all samples were 25 mg/mL. Then, they were diluted with different buffers). Then, the mixtures were incubated at 50 °C for 20 min. Later, 2.5 mL of trichloroacetic acid (TCA) (10%, *w*/*v*) was added to the mixture to stop the reaction. Finally, 1.5 mL of FeCl_3_ (0.1%, *w*/*v*) was added, and the absorbance of the solutions at 700 nm was measured.

#### 4.5.4. DPPH Radical Scavenging Activity

The DPPH free radical assay was based on a modified method [30]. Briefly, 1 mL of 0.1 mmol L^−1^ DPPH solution in ethanol was added to the solution (3 mL) with different concentrations of samples (dissolved in 50% ethanol). Firstly, the original concentrations of all samples were 25 mg/mL. Then, they were diluted with different buffers. The mixtures were then shaken vigorously and allowed to stand at room temperature for 20 min. The absorbance at 517 nm was measured. As a control, the 50% ethanol solution was substituted for sample. The scavenging effect (percentage) was calculated with the following equation: Scavenging effect (%) = (1 − A_sample_/A_control_) × 100.

### 4.6. Statistical Analysis

All data are shown as the mean ± standard deviation (SD). Significant differences between experimental groups were determined by one-way ANOVA, and differences were considered as statistically significant if *p* < 0.05. All calculations were performed using SPSS 16.0 statistical Software.

## 5. Conclusions

ESI-MS with ESI-CID-MS/MS was performed to elucidate the structure of SGs. However, this technique failed because there were an insufficient number of A-type or X-type ions to indicate the linkages of the sulfation pattern. Therefore, NMR was performed, and this technique showed that the order of sulfation was Man-C6 > Man-C4 > Man-C1R > GlcA-C3 > Man-C3 > GlcA-C2. The antioxidant activities of ten sulfated oligosaccahrides in four assays, i.e., hydroxyl radical scavenging activity, superoxide radical scavenging activity, reducing power and DPPH radical scavenging activity, were determined. It was shown that sulfate content and degree of polymerization represented different degrees of effects on the antioxidant activities. In addition, compared with fucoidan, most Gs and SGs molecules had higher activities in terms of hydroxyl radical scavenging activity, reducing power and DPPH radical scavenging, although superoxide radical scavenging activity was different. In conclusion, Gs and SGs might be good candidates for anti-oxidants.

## Figures and Tables

**Table 1:** The chemical compositions of glucuronomannan oligosaccharides and their sulfated fractions.

Sample	Chemical Compositions
G2S1	GlcAMan(SO_3_H)_3–6_
G2S2	GlcAMan(SO_3_H)_1–3_
G2	GlcAMan
G4S1	GlcA_2_Man_2_(SO_3_H)_8–11_
G4S2	GlcA_2_Man_2_(SO_3_H)_5–9_
G4S3	GlcA_2_Man_2_(SO_3_H)_1–5_
G4	GlcA_2_Man_2_
G6S1	GlcA_3_Man_3_(SO_3_H)_8_–_15_
G6S2	GlcA_3_Man_3_(SO_3_H)_4–10_
G6S3	GlcA_3_Man_3_(SO_3_H)_1–6_
G6	GlcA_3_Man_3_

## Supplementary Materials

The following are available online at http://www.mdpi.com/1660-3397/16/9/291/s1, Figure S1: Negative-ion mode ESI-MS spectrum of G2S1, Figure S2: Negative-ion mode ESI-MS spectrum of G2S2, Figure S3: Negative-ion mode ESI-MS spectrum of G4S1, Figure S4: Negative-ion mode ESI-MS spectrum of G4S2, Figure S5: Negative-ion mode ESI-MS spectrum of G4S3, Figure S6: Negative-ion mode ESI-MS spectrum of G6S1, Figure S7: Negative-ion mode ESI-MS spectrum of G6S2, Figure S8: Negative-ion mode ESI-MS spectrum of G6S3, Figure S9: The DEPTQ spectra of a glucuronomannan tetramer (G4), its low sulfated fraction (G4S3) and its medium sulfated fraction (G4S2), Table S1: The chemical shifts of G4 and G6.
