# Supplementary material for: Structural Features of Sulfated Glucuronomannan Oligosaccharides and Their Antioxidant Activity
Source: Mar Drugs. 2018 Aug 21;16(9):291. doi: 10.3390/md16090291 (PMC6165275; doi:10.3390/md16090291)
Supplement: Supplementary file 1 [file marinedrugs-16-00291-s001.pdf]

Supplementary data

Table S1 The chemical shifts of G4 and G6

Samples	Residues	C1	C2	C3	C4	C5	C6	H1	H2	H3	H4	H5	H6
G4	→2)-α-D-Manp-OH	92.5	78.6	69.8	67.4	72.7	60.8	5.14	3.91	3.73	3.54	3.58	3.63-3.68
	→4)-β-D-GlcAp-(1→	102.0	73.0	76.7	77.2	76.5	175.4	4.37	3.23	3.52	3.62	3.66	-
	→2)-α-D-Manp-(1→	98.8	77.9	69.9	66.8	72.9	60.4	5.28	4.05	3.71	3.58	3.58	3.63-3.68
	β-D-GlcAp-(1→	101.8	73.1	75.6	72.2	76.2	176.2	4.33	3.28	3.38	3.62	3.64	-
G6	→2)-α-D-Manp-OH	92.5	78.6	69.8	67.4	72.7	60.9	5.14	3.91	3.71	3.52	3.58	3.64-3.68
	→4)-β-D-GlcAp-(1→	102.0	73.0	76.5	77.2	76.8	175.4	4.36	3.26	3.52	3.63	3.64	-
	→2)-α-D-Manp-(1→	98.8	77.8	69.9	66.8	72.9	60.4	5.27	4.02	3.71	3.58	3.58	3.64-3.68
	→4)-β-D-GlcAp-(1→	101.8	73.1	76.4	77.1	76.7	175.5	4.33	3.26	3.52	3.63	3.64	-
	→2)-α-D-Manp-(1→	98.8	78.0	69.9	66.9	73.0	60.4	5.27	4.05	3.71	3.58	3.58	3.64-3.68
	β-D-GlcAp-(1→	101.8	73.1	75.6	72.2	76.3	176.3	4.33	3.28	3.38	3.62	3.64	-

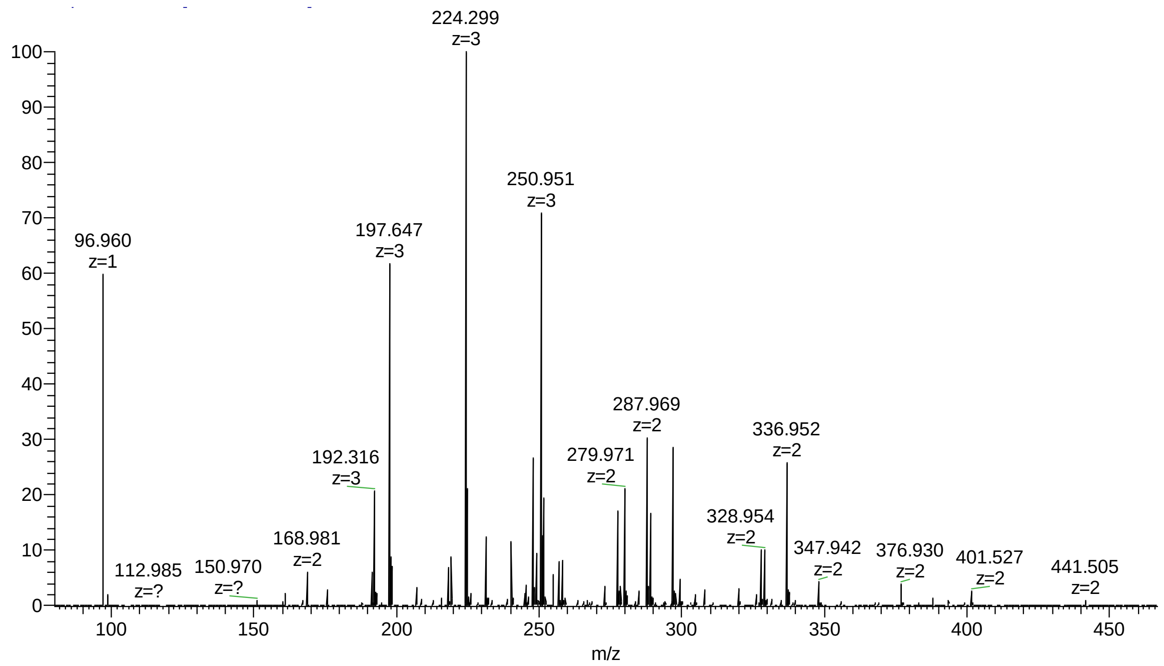

Figure S1 Negative-ion mode ESI-MS spectrum of G2S1.

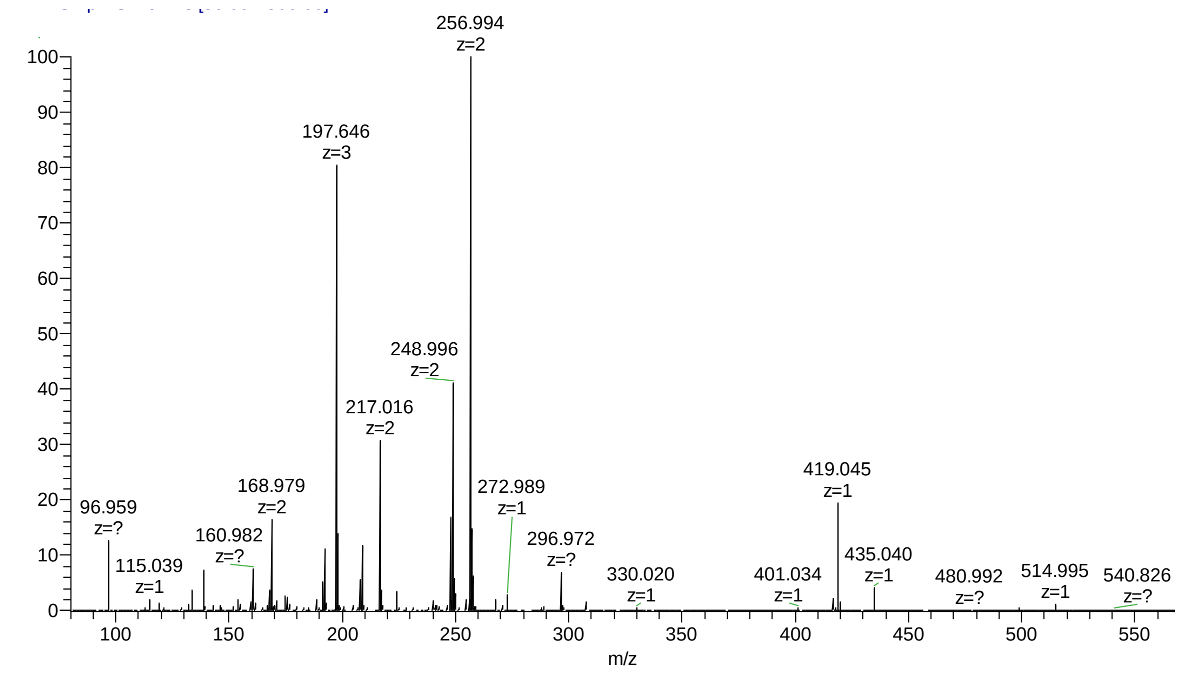

Figure S2 Negative-ion mode ESI-MS spectrum of G2S2.

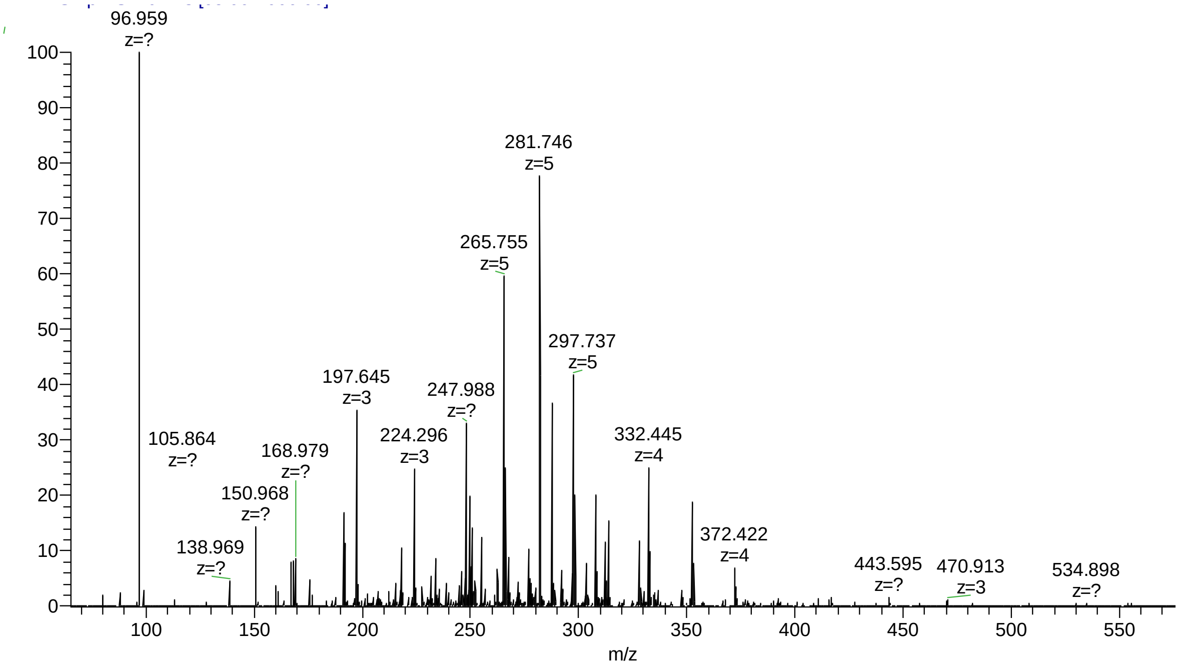

Figure S3 Negative-ion mode ESI-MS spectrum of G4S1.

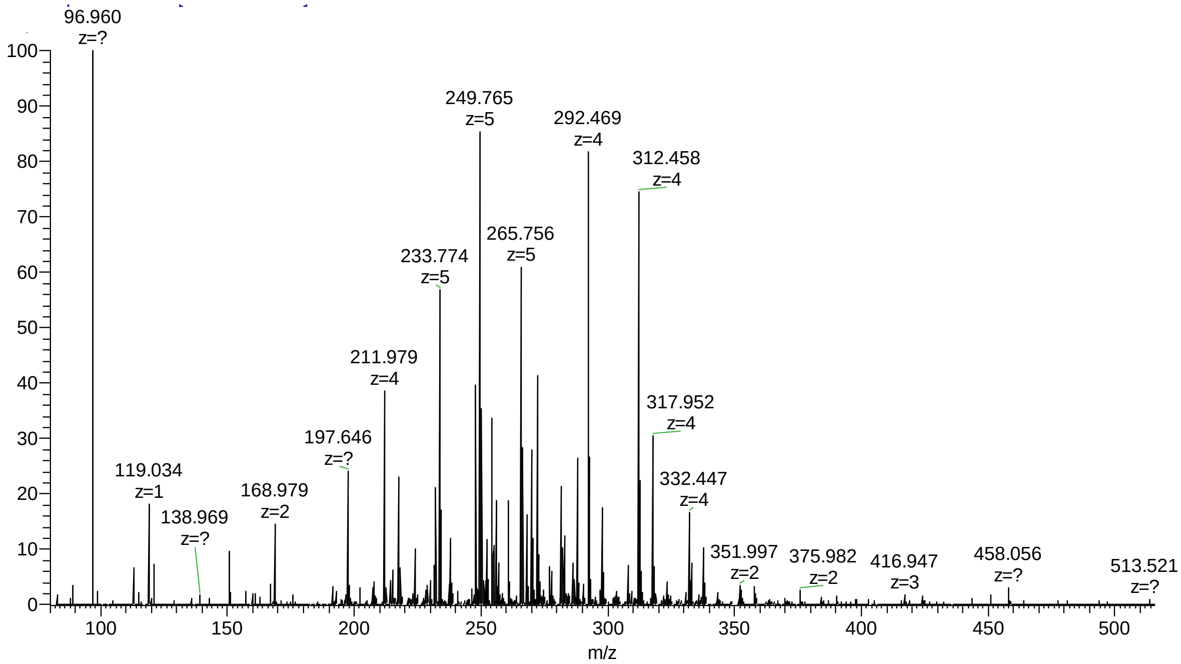

Figure S4 Negative-ion mode ESI-MS spectrum of G4S2.

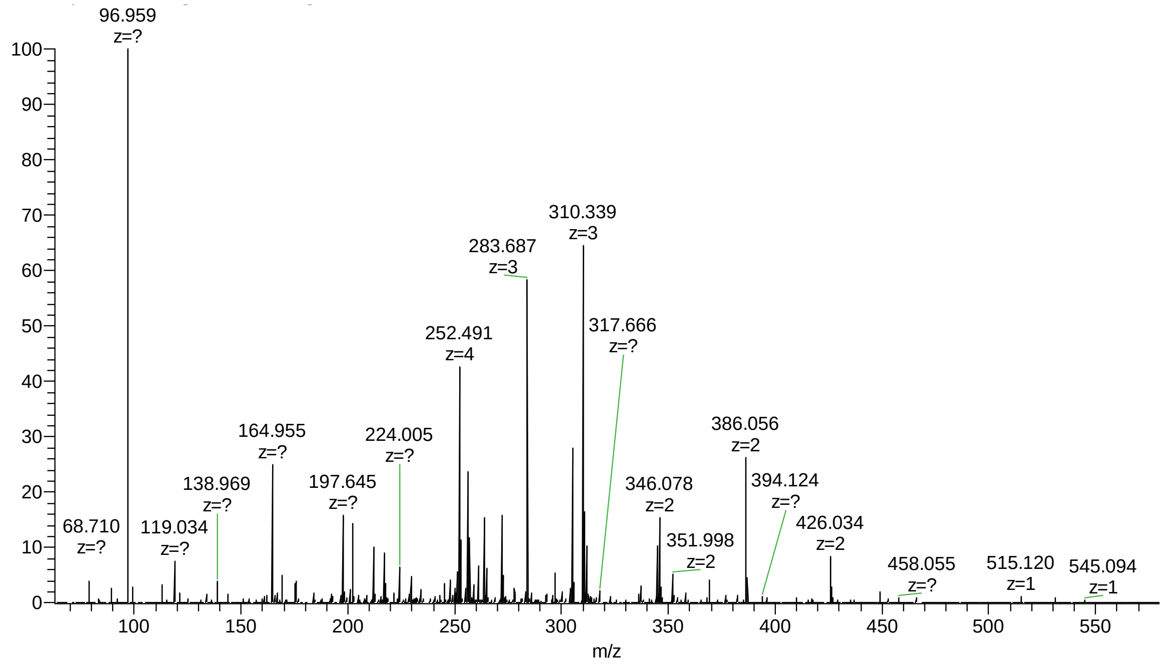

Figure S5 Negative-ion mode ESI-MS spectrum of G4S3.

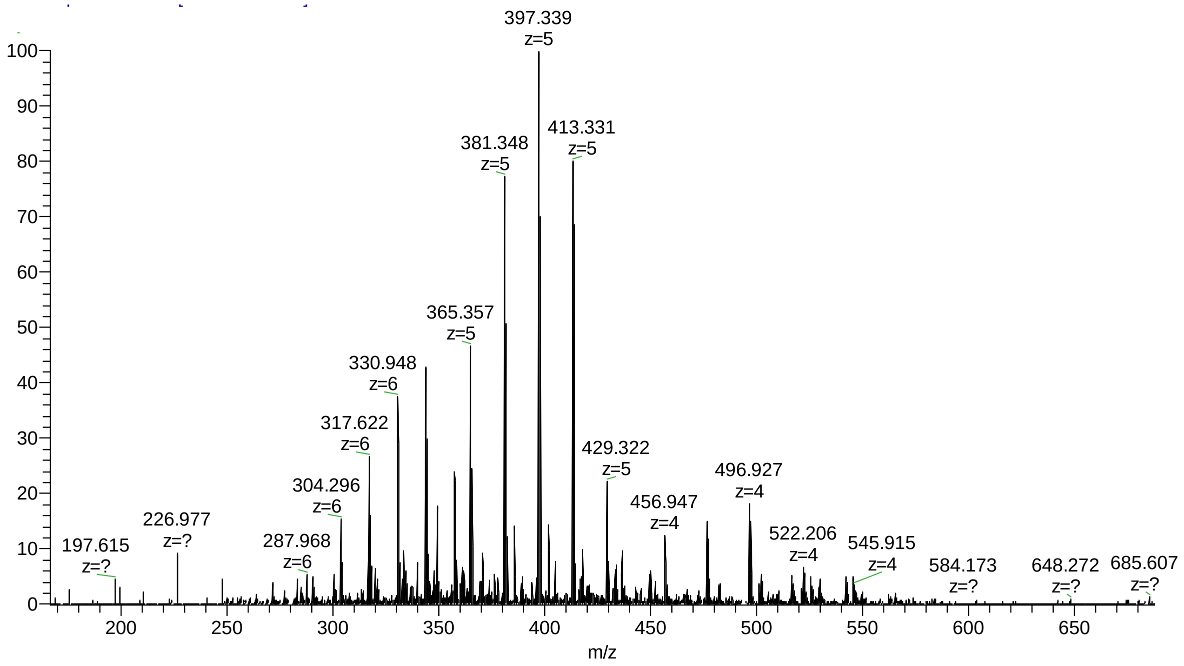

Figure S6 Negative-ion mode ESI-MS spectrum of G6S1.

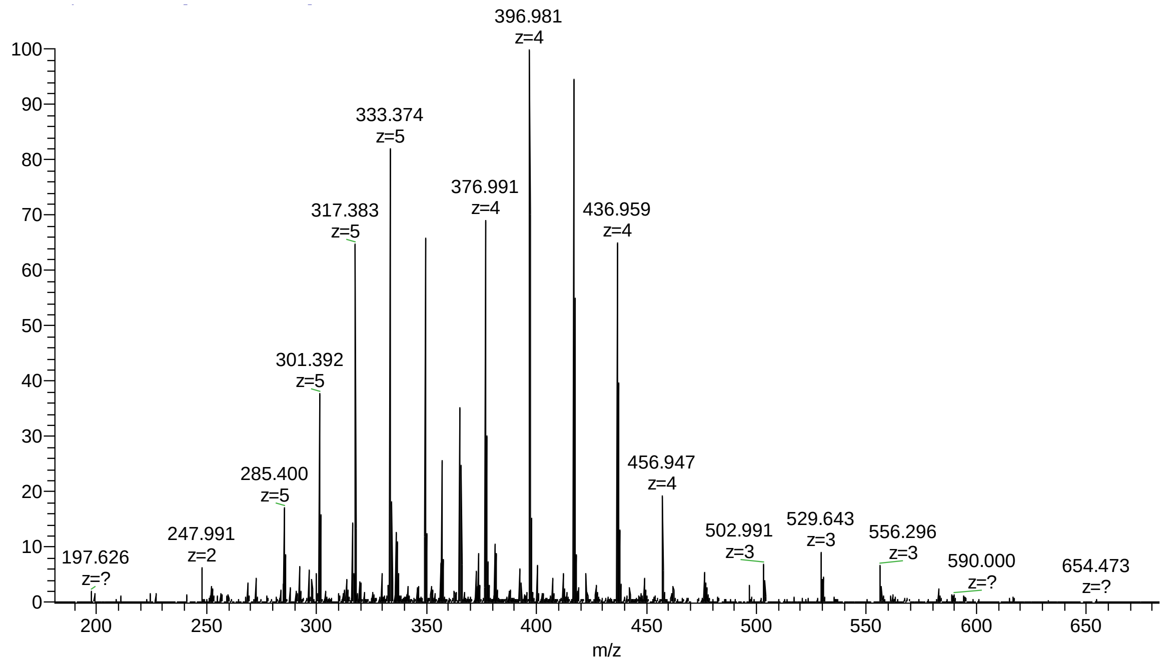

Figure S7 Negative-ion mode ESI-MS spectrum of G6S2.

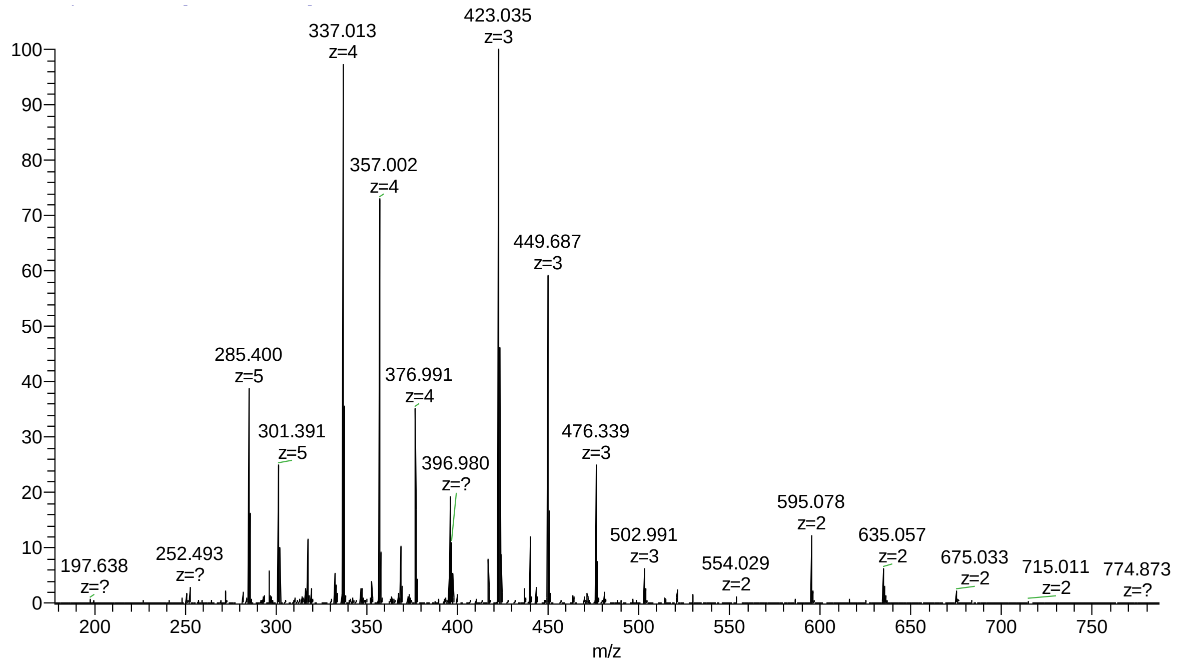

Figure S8 Negative-ion mode ESI-MS spectrum of G6S3.

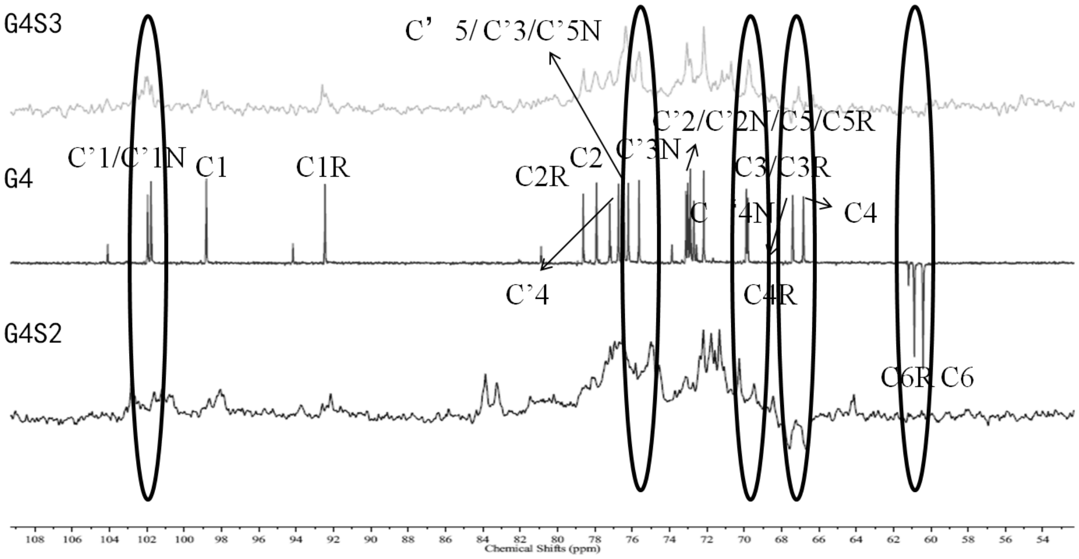

Fig. S9 The DEPTQ spectra of a glucuronomannan-tetramer (G4), its low sulfated fraction (G4S3) and its medium sulfated fraction (G4S2).
